# Multi-Drug Resistant Organisms Infection Impact on Patients Length of Stay in Respiratory Care Ward

**DOI:** 10.3390/antibiotics10050608

**Published:** 2021-05-20

**Authors:** Yi-Ping Chen, Xian-Wen Tasi, Ko Chang, Xuan-Di Cao, Jung-Ren Chen, Chien-Sen Liao

**Affiliations:** 1 Department of Medical Laboratory, Kaohsiung Municipal Siaogang Hospital, Kaohsiung Medical University, Kaohsiung 81267, Taiwan; 880475@kmuh.org.tw; 2 Department of Nursing and Medical Quality Management Center, Kaohsiung Municipal Siaogang Hospital, Kaohsiung Medical University, Kaohsiung 81267, Taiwan; 910139@kmuh.org.tw; 3 Department of Internal Medicine, Kaohsiung Municipal Siaogang Hospital, Kaohsiung Medical University, Kaohsiung 81267, Taiwan; johnsonckk@yahoo.com.tw; 4 College of Medicine, Kaohsiung Medical University, Kaohsiung 80708, Taiwan; 5 Institute of Biotechnology and Chemical Engineering, I-Shou University, Kaohsiung 84001, Taiwan; isu10937051d@cloud.isu.edu.tw; 6 Department of Biological Science and Technology, I-Shou University, Kaohsiung 82445, Taiwan

**Keywords:** length of stay, multi-drug resistant organisms, respiratory care ward, carbapenem-resistant *Pseudomonas aeruginosa*

## Abstract

This study aimed to investigate the effects of multi-drug-resistant organism (MDRO) infection and other factors on the length of hospital stay (LOS) of patients in the respiratory care ward (RCW) of a regional hospital in Taiwan. In this retrospective study, we collected cases from MDRO-infected patients in the RCW from January 2016 to March 2020. The RCW comprises 13 beds in total. There were 106 infected patients, of which 42 were in the case group (infected with MDROs) and 64 were in the control group (not infected with MDROs). Clinical specimens were inoculated in a selective medium to isolate the pathogenic bacteria by standard procedures. The results showed the main factors affecting the LOS were: patients with MDRO infection, patients discharged from the RCW, and patients who underwent catheterization. The LOS of patients infected with MDROs was significantly longer than that of patients without MDRO infection (β = 0.55, 95% CI = 0.02–1.09), with the case group and the control group being 479.8 ± 546.5 and 307.3 ± 436.2 days, respectively. Infection with carbapenem-resistant *Pseudomonas aeruginosa* (CRPA) was associated with a longer LOS than other MDRO strains. These findings have important implications for infection control in RCW and in better tracking the health of patients.

## 1. Introduction

Airway protection in patients with respiratory distress requires intubation and ventilation support via an artificial airway. During intubation, it is easy to damage the natural defense mechanisms of the oropharynx, causing bacterial infection of the lower respiratory tract, leading to ventilator-associated pneumonia (VAP) [1,2]. VAP is a serious complication that significantly impacts the prognosis of patients in RCWs, and it incurs additional medical expenses [3]. Repeated cases of VAP have been documented in critically ill patients having undergone endotracheal intubation and ventilation apparatus support in RCWs [4]. These patients require a variety of invasive interventions, and the long-term hospital stay has a huge impact on medical costs. Moreover, antibiotics that are widely used in order to avoid infections in patients drive the generation of multi-drug-resistant organisms (MDROs): pathogenic bacteria resistant to more than one kind of antibiotic, which ultimately lead to poor therapeutic control by antibiotics [5,6].

Healthcare-associated infections (HAIs) also extend the total length of stay in the hospital. According to a study by Jia et al. [7], HAIs increased the economic burden to patients in 68 hospitals in China. This difference was statistically significant (*p* < 0.01). According to estimates by Zilahi et al. [8], HAIs in the ICU in Iran resulted in a relatively hefty financial burden related to antibiotics, higher mortality rates, and longer hospital stays. The extra hospital stay for bloodstream infections (BSIs) was 3.48 days, urinary tract infections (UTIs) was 3.59 days, surgical site infections (SSIs) was 7.23 days, and VAP was 11.52 days. One study also reported variable LOS for different infection sites: LOS of central line-associated bloodstream infections (CLABSI), ventilator-associated pneumonia (VAP), and catheter-associated urinary tract infections (CAUTI) were 27.1, 22.2, and 19.2 days, respectively [9].

MDROs among deceased organ donors are a risk factor for medical-related infections [10] and one of the risk factors for prolonging LOS. Infection control measures to reduce cross-spread should include strategies to decrease the infection rate of various parts of the RCW [11], reduce overuse of medical resources, and reduce LOS, thereby optimizing antibiotic management [12].

The objectives of this study were to investigate the effects of MDRO infection and other factors on the LOS of patients in the RCW of a regional hospital in Taiwan. These results have important implications for infection control in the RCW and may help with devising prevention and control strategies for multi-drug-resistant bacterial infections. These data may also provide insight regarding the health of patients and clinical medical staff in high-acuity medical units through improved infection information.

## 2. Materials and Methods

### 2.1. Research Design and Data Collection

This study was conducted in a regional teaching hospital with 496 patient beds. This hospital is an important tertiary care medical institution in Kaohsiung, Taiwan. The present study is a retrospective review of hospital-acquired infections due to MDRO strains collected from patients in the RCW from January 2016 to March 2020. The RCW comprises 13 beds in total. The positivity rates of MDRO-infected patients over the five-year period were analyzed by WHONET. Inclusion criteria targeted only patients identified as having their first confirmed infection of a MDRO strain in a given year. A total of 42 MDRO-infected patients and 64 control patients were enrolled in the study. Ethical approval for this study was obtained from the Institutional Review Board, Kaohsiung Medical University, Kaohsiung, Taiwan, approval number KMUHIRB-E(I)-20190148.

### 2.2. Antibiotic Susceptibility and Species Identification of Bacterial Isolates

Clinical specimens were inoculated in a selective medium to isolate the pathogenic bacteria by standard procedures. Bacterial isolates were speciated, and antibiotic susceptibility was determined on automated VITEK and VITEK^®^2 Compact platforms (bioMérieux, Inc., Hazelwood, MO, USA). Full traceability and minimization of transcription errors were ensured using automated bar-coding technology. VITEK^®^2 Compact GN (bioMérieux, VITEK 2 AST-N339 REF419341, Marcy-l’Étoile, France) was used for identifying CRE, CRAB, and CRPA. Compact GP (bioMérieux VITEK 2 AST-P627 REF414124, Marcy-l’Étoile, France) was used for identifying vancomycin-resistant *Enterococcus faecium* (VREfm) and MRSA. Antibiotic susceptibility was based on the definitions of the 2016 version of CLSI (Clinical and Laboratory Standards Institute, Wayne, PA, USA). ATCC25922, ATCCBAA-1705, and ATCCBAA-170 were used as standard strains. The culture medium was provided by Coning Technology Limited Company (Taichung, Taiwan). The minimum inhibitory concentration (MIC) of antibiotic susceptibility was determined according to the MCN-6 system (Merlin, Diagnostics, Bornheim-Hersel, Germany) and Clinical and Laboratory Standards Institute (CLSI, Wayne, PA, USA) specifications. *E. coli* ATCC 25922, *E. coli* ATCC 35218, and *K. pneumoniae* ATCC 700603 were used as positive and negative control strains.

### 2.3. Definition and Data Collection of Risk Factors

Multi-drug-resistant organisms (MDROs) are pathogenic bacteria resistant to more than one kind of antibiotic. This kind of bacteria is usually resistant to many kinds of antibiotics, and often only one or two of the existing antibiotics are effective. Common MDROs include carbapenem-resistant *Acinetobacter baumannii* (CRAB), carbapenem-resistant *Klebsiella pneumoniae* (CRKP), CRPA, methicillin-resistant *Staphylococcus aureus* (MRSA), methicillin-resistant *Staphylococcus epidermidis* (MRSE), and vancomycin-resistant *Enterococcus faecium* (VREfm).

Data were collected from the charts of infected patients to identify risk factors, including sex (male, female), age (≤18, 19–60, and ≥61 years old), types of specimens (sputum, urine, abscess, blood, and others), cultured antibacterial-drug-resistant strains (vancomycin-resistant *Enterococcus* (VRE), carbapenem-resistant *Escherichia coli* (CREC), MRSA, CRE, CRKP, CRAB, and CRPA), cultured antimicrobial susceptibility tests (non-controlled or controlled drugs), use of catheters (i.e., endoscopes, urinary catheters, ventilators, central venous catheters, etc.), and the logarithm of the number of days from admission to discharge (or death). Risk factors were categorized and analyzed. Length of hospital stay (LOS) was defined as the number of days from admission to discharge (or death), and was the primary outcome variable of this study. The classification of the medications used by patients before MDRO infection was as follows: anti-Gram-positive bacteria drugs were erythromycin, moxifloxacin, and amp/sulbactam; anti-Gram-negative bacteria drugs were cefmetazole and amp/sulbactam; anti-*Pseudomonas aeruginosa* drugs were cefoperazole/sulbactam, ceftazidime, cefepime, piperacillin/tazobactam, meropenem, imipenem, levofloxacin, ciprofloxacin, colistin, and amikacin; and MRSA drugs were vancomycin, teicoplanin, tigecycline, daptomycin, and linezolid.

### 2.4. Statistical Analysis

The data collected were compiled and analyzed with Excel 2016 and SPSS statistical software version 24.0 (IBM, New York, NY, USA). Descriptive statistical analysis was used between variables, and LOS was analyzed using the Mann–Whitney U-test [13,14], one-way ANOVA, and post hoc testing. The relationship between the number of days in hospital and the variables was analyzed by multivariate regression analysis (two-way ANOVA). The significance threshold was set to 0.05.

## 3. Results

### 3.1. Research Design and Data Collection

**Figure 1:**
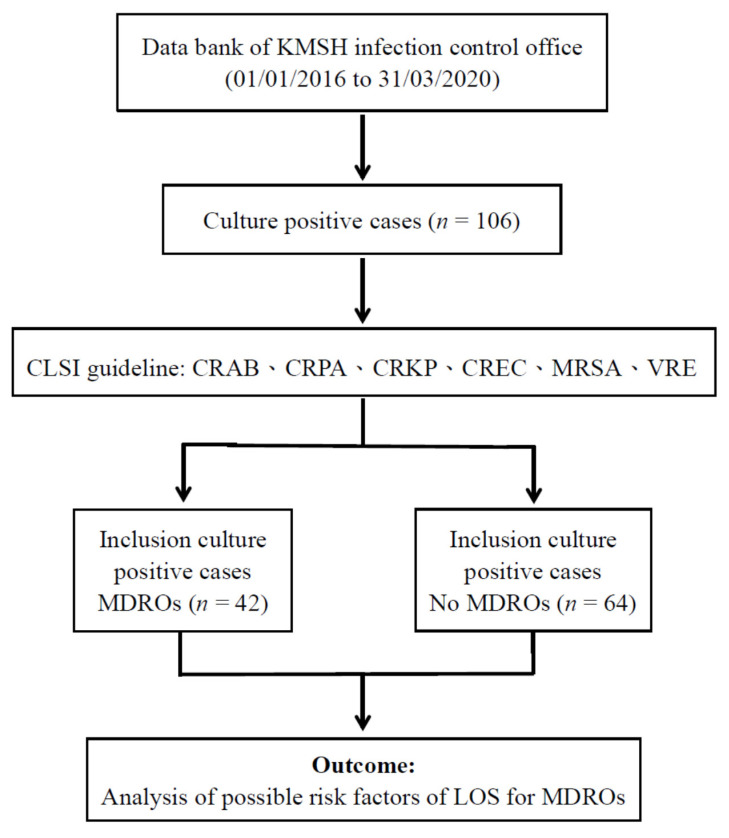
Experimental flow chart.

As shown in Figure 1, during the study period, a total of 106 cases were collected and included in this study. Further analysis showed that 42 cases (39.63%) were MDROs (case group), and the number of non-MDRO cases was 64 (60.37%) (control group). The average age of patients was 73.21 years old, and 62.0% of them were men. The first objective of this research was to analyze the risk factors related to HAI in the RCW. The second objective was to conduct an association study between LOS and RCW MDROs.

### 3.2. Case Analysis of MDRO-Infected Patients

**Figure 2:**
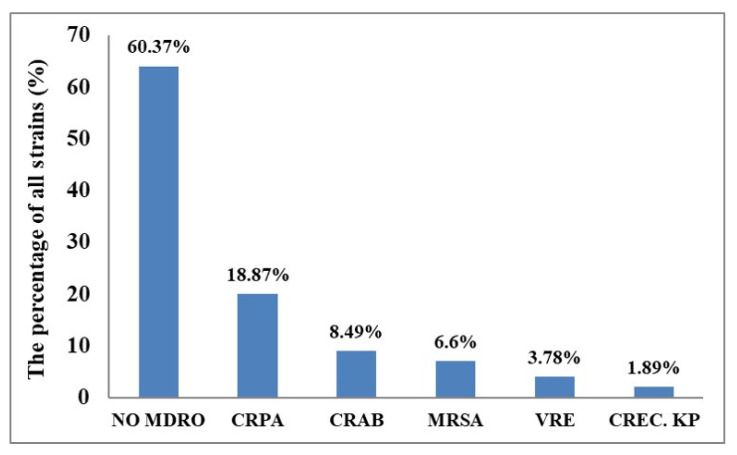
Strain percentages comprising all HAI.

Figure 2 shows that there were 106 cases of infection in the RCW: 42 patients with MDROs (case group) and 64 patients without MDROs (control group, 60.37%). MDROs included 20 strains of CRPA (18.87%), 9 strains of CRAB (8.49%), 7 strains of MRSA (6.6%), 4 strains of VRE (3.78%), and 2 strains of CREC. KP (1.89%). Comparing the infections of different MDRO strains, we found that CRPA strains accounted for about 47.62% of MDRO strains. In a post hoc analysis, we determined that the LOS of MDRO-infected patients (479.8 ± 546.5 days) was longer than that of the control group (307.3 ± 436.2 days). The LOS of patients without any bacterial infection was 121.3 ± 65.9 days (Management office, Kaohsiung Municipal Siaogang Hospital (KMSH), from January 2016 to March 2020). This difference is statistically significant (*p* = 0.018).

### 3.3. Risk Factor Data Collection

Patient demographic information is shown in Table 1, and includes: age, sex, previous ward, type of ward when discharged, last departure, nutritional score, antibiotic use, controlled drugs, anti-*PsA* drugs, anti-MRSA drugs, use of catheters, use of endoscopes, use of CVC, use of FOLEY, use of respirators, and LOS. The results showed that the use of anti-MRSA drugs may increase the production of and infection with MDROs compared with anti-*PsA* drugs (*p* = 0.006). We found that the average LOS in the MDRO group was 479.8 ± 546.5 days, resulting in a mean additional LOS of 172 more days than that of the non-MDROs group (307.3 ± 436.2, *p* = 0.018). There was no statistically significant difference in the other related risk factors (*p* > 0.05).

### 3.4. Factors Affecting the Length of Hospital Stay

As shown in Table 2, we explored factors affecting the LOS in terms of demographic variables and clinical treatment characteristics with statistically significant influence of age and MDRO status.

#### 3.4.1. Analysis of Variance (One-Way ANOVA) 

The statistical results showed that the main factors affecting the LOS in the RCW were: presence of MDRO infection (β = 0.62, 95% CI = 0.05–1.18, *p* = 0.033), age (β = 0.68, 95% CI = 0.10–1.27, *p* = 0.023), having left the nursing station in the RCW last (β = 1.52, 95% CI = 0.94–2.11, *p* < 0.0001), use of catheters (β = 1.37, 95% CI = 0.07–2.04, *p* < 0.0001), and use of a respirator (β = 0.99, 95% CI = 0.46–1.53, *p* < 0.0001). There were no statistically significant differences in the other variables (*p* > 0.05).

#### 3.4.2. Multivariate Regression Analysis (Two-Way ANOVA)

The data from Section 3.4.1 were also subjected to multivariate analysis (Table 2). The main factors influencing the LOS included patients with MDRO infection, patients who left the nursing station in the RCW, and patients who underwent catheterization. Patients with MDRO infection had significantly greater LOS than those without MDRO infection (β = 0.55, 95% CI = 0.02–1.09, *p* = 0.037). The LOS of patients who were discharged from the RCW was significantly longer than the LOS of patients discharged from the general ward (β = 1.16, 95% CI = 0.52–1.80, *p* < 0.001). There was significantly greater LOS in patients with catheters than without catheters (β = 0.86, 95% CI = 0.03–1.70, *p* = 0.043). There were no statistically significant differences between the other variables (*p* > 0.05).

### 3.5. Analysis of Variance (ANOVA) of log LOS of Different Drug-Resistant MDROs

As shown in Table 3, a one-way ANOVA found that there was a significant difference between the log LOS for bacteria with different MDROs (*p* < 0.018). Post hoc testing using log LOS as the dependent variable comparing different MDROs is presented in Table 4. The results showed that log LOS was significantly different between CRPA and CRAB of MDROs (*p* < 0.037). Scheffe’s post hoc test found that the log LOS of CRPA was greater than CRAB, representative of the strains of MDROs in the RCW during the research period. In other words, the CRPA and CRAB of MDROs affected the difference in log LOS days (*p* < 0.037 *). CRPA was associated with a longer LOS than other strains of MDROs. The other strains of MDROs did not affect the increase in LOS, and there was no statistically significant difference (*p* > 0.005). Further analysis of these MDRO infections in RCW revealed that the average LOS increase was 158.90 days. Comparing different MDRO infections, we found that CR-PA-induced LOS was an average of 643.30 days, which is longer than that of other MDRO infections. For example, the average LOS of VRE was 112.00 days, the average LOS of MRSA was 484.86 days, and the average LOS of CREC. KP was 415.50 days. The CRAB average LOS was 272.40 days.

## 4. Discussion

### 4.1. MDRO Infection Is a Risk Factor for Prolonged LOS

The present study focused on LOS related to infection by MDROs, because patients in the RCW tend to be older, immunocompromized, and have catheter-related issues. The LOS in the RCW is longer than that of the outpatient and emergency departments or general and surgical wards. For example, the LOS for patients with MDROs such as CRAB and CRPA is significantly longer, as evidenced by statistical analysis (*p* < 0.037). Taking a similar approach to one study comparing different MDRO infections in mainland China [7], we found that the increase in LOS associated with HAI due to CRPA was significantly longer than other MDRO infections, other than VRE and CR-*E. coli* infection (*p* < 0.05). There was no significant difference between other MDRO infections (*p* > 0.05).

In line with the findings of several previous studies [15,16,17], the present study also found the increase in LOS of HAI to be more than that of uninfected patients. This evidence suggests that MDROs are a risk factor for prolonged LOS.

### 4.2. Risk Factors Affecting LOS

The risk factors for MDRO infection are well-documented: (1) patients with a history of hospitalization for more than 2 days in the past 90 days; (2) patients in nursing homes or long-term care centers; (3) patients who received antibiotic treatment in the past 90 days [18]. Among hospitalized patients in Athens, Greece, long-term hospitalization and age were independent risk factors for carrying VRE, while infection with CRGN was associated with an increased risk of acquiring drug-resistant pathogens, prolonged hospital stays, and increased mortality [19]. Patients suffering from ICU-acquired paresis (ICUAP) due to MDROs have a higher ICU mortality rate than non-MDRO patients. Patients without microbiological confirmation are more often treated with antibiotics than those with positive cultures [20]. In addition to local epidemiology, the risk factors of MDROs may also be important for the choice of initial antibacterial therapy. We found that the univariate risk factors associated with LOS were MDROs (yes vs. none), age (>65 years vs. other), use of catheters (yes vs. none), respirators (yes vs. none), and final discharge station status (severe vs. general). The analysis of multivariate ANOVA found that MDROs (yes vs. no), use of catheters (yes vs. no), and final departure (severe vs. general) affected the LOS. These results are slightly different from previous studies [18,21]. Further studies are needed to settle this discrepancy.

Our results show the main factors affecting LOS in patients are MDRO infection, hospitalization in the RCW, and catheterization. The results of this study provide relevant data to supplement existing medical care-related infection data and provide insights into how to better maintain the health of patients and clinical medical staff through the exchange of infection information related to the RCW. Our study underscores the considerable health burden of MDRO infections and addresses the urgent need for improved antimicrobial stewardship to improve public health in Taiwan. 

### 4.3. Advantages

The advantages of the present research are: (1) there was a paucity of current literature addressing specific analysis of MDROs in RCW; (2) risk factors impacting LOS have not been previously analyzed with such rigor in the RCW hospital care setting in Taiwan.

### 4.4. Limitations

The present study only shows that the presence of MDROs is a risk factor for prolonged LOS. In order to establish a causal relationship between MDRO infection and LOS, we would have needed to take an approach similar to the study by Barrasa-Villar (2017) who looked only at LOS after diagnosis of MDRO infection and controlled for hospital LOS prior to the infection. However, the causal relationship between MDRO infection and LOS was beyond the scope of their investigation.

This study is also limited by being conducted in a single regional hospital, with a limited number of cases and MDRO strains, and the time encompassed was limited to a 5-year period. If coordinated data can be collected from more hospitals with relevant MDRO strains in RCW patients, and pertinent clinical data can be provided for further analysis, this would allow a more representative and comprehensive conclusion to be drawn and be applied to more hospitals.

## 5. Conclusions

We investigated the effects of MDRO infection and other factors on the LOS of patients in the RCW of a regional hospital in Taiwan. The results showed that infection by multi-drug-resistant organisms impacted the LOS of patients in the RCW, and that CRPA strains were the most common MDROs in the RCW, comprising 47.62% of MDRO infections. ANOVA and post hoc testing of log LOS found that different MDROs, specifically CRPA and CRAB, have a significant impact on log LOS days (*p* < 0.037). The main factors affecting LOS achieving statistical significance (*p* < 0.05) were MDRO infection, hospitalization in the RCW, and catheterization. The findings of this study provide useful information for the hospital RCW about the impact of MDROs on LOS of patients. These findings may also improve the level of clinical medical care and provide holistic health care service strategies, which can be used to improve infection control in the future.

## Figures and Tables

**Table 1:** Descriptive statistical analysis of risk factors.

Variable	Overall (*n* = 106)	Non-MDROs (*n* = 64)	MDROs (*n* = 42)	*p*-Value
	*n* (%)/Mean ± SD	*n* (%)/Mean ± SD	*n* (%)/Mean ± SD	
Age (years)				
>65	71 (67.0)	40 (62.5)	31 (73.8)	0.226
other	35 (33.0)	24 (37.5)	11 (26.2)	
Sex				
Male	55 (51.9)	32 (50.0)	23 (54.8)	0.631
Female	51 (48.1)	32 (50.0)	19 (45.2)	
Sample				
Sputum	47 (44.3)	24 (37.5)	23 (54.8)	0.110
Urine	31 (29.2)	24 (37.5)	7 (16.7)	
Blood	16 (15.1)	10 (15.6)	6 (14.3)	
Other	12 (11.3)	6 (9.4)	6 (14.3)	
Previous ward				
None	65 (61.3)	39 (60.9)	26 (61.9)	0.920
Yes	41 (38.7)	25 (39.1)	16 (38.1)	
Type of ward discharged				
General ward	26 (24.5)	17 (26.6)	9 (21.4)	0.548
RCW	80 (75.5)	47 (73.4)	33 (78.6)	
Nutritional score	2.2 ± 1.2	2.1 ± 1.2	2.1 ± 1.2	0.407
Antibiotics				
None	20 (18.9)	15 (23.4)	5 (11.9)	0.138
Yes	86 (81.1)	49 (76.6)	37 (88.1)	
Controlled drugs				
None	24 (22.6)	18 (28.1)	6 (14.3)	0.096
Yes	82 (77.4)	46 (71.9)	36 (85.7)	
Anti- *PsA* drugs				
None	32 (30.2)	23 (35.9)	9 (21.4)	0.111
Yes	74 (69.8)	41 (64.1)	33 (78.6)	
Anti-MRSA drugs				
None	65 (61.3)	46 (71.9)	19 (45.2)	0.006 *
Yes	41 (38.7)	18 (28.1)	23 (54.8)	
Catheterization				
None	20 (18.9)	13 (20.3)	7 (16.7)	0.639
Yes	86 (81.1)	51 (79.7)	35 (83.3)	
Use of endoscope				
None	67 (63.2)	40 (62.5)	27 (64.3)	0.852
Yes	39 (36.8)	24 (37.5)	15 (35.7)	
Use of CVC				
None	90 (84.9)	55 (85.9)	35 (83.3)	0.714
Yes	16 (15.1)	9 (14.1)	7 (16.7)	
Use of FOLEY				
None	61 (57.5)	38 (59.4)	23 (54.8)	0.638
Yes	45 (42.5)	26 (40.6)	19 (45.2)	
Use of ventilator				
None	47 (44.3)	29 (45.3)	18 (42.9)	0.803
Yes	59 (55.7)	35 (54.7)	24 (57.1)	
LOS ^a^	375.6 ± 487.9	307.3 ± 436.2	479.8 ± 546.5	0.018 *

*: The mean difference is significant at the 0.05 level. ^a^: Logarithm of the number of days in hospital.

**Table 2:** Factors affecting the length of hospital stay.

Variable	Univariate		Multivariate	
	β (95% CI)	*p*-Value	β (95% CI)	β (95% CI)
MDROs (Yes vs. None)	0.62 (0.05, 1.18)	0.033 *	0.55 (0.02, 1.09)	0.037 *
Age (>65 years vs. other)	0.68 (0.10, 1.27)	0.023 *	0.25 (−0.34, 0.84)	0.410
Sex (Female vs. Male)	−0.36 (−0.92, 0.20)	0.207	−0.05 (−0.60, 0.50)	0.857
Sample				
Other	Ref.		Ref.	
Sputum	0.81 (−0.12, 1.74)	0.087	0.86 (−0.01, 1.73)	0.052
Urine	0.48 (−0.50, 1.46)	0.331	0.82 (−0.17, 1.81)	0.102
Blood	1.01 (−0.09, 2.11)	0.071	1.12 (0.11, 2.14)	0.031
Previous ward (Yes vs. None)	0.002 (−0.58, 0.58)	0.993	0.26 (−0.35, 0.86)	0.403
Type of ward discharged (RCW vs. General ward)	1.52 (0.94, 2.11)	<0.001 *	1.16 (0.52, 1.80)	<0.001 *
Nutritional score	0.16 (−0.08, 0.40)	0.196	−0.06 (−0.30, 0.18)	0.616
Antibiotics (Yes vs. None)	0.59 (−0.12, 1.30)	0.104	0.31 (−0.66, 1.28)	0.530
Controlled drug (Yes vs. None)	0.60 (−0.07, 1.26)	0.077	−0.03 (−1.48, 1.42)	0.966
Anti-*PsA* drugs (Yes vs. None)	0.52 (−0.09, 1.13)	0.093	0.11 (−0.99, 1.21)	0.844
Anti-MRSA drugs (Yes vs. None)	0.19 (−0.38, 0.77)	0.507	−0.28 (−0.93, 0.37)	0.400
Catheterization (Yes vs. None)	1.37 (0.70, 2.04)	<0.001 *	0.86 (0.03, 1.70)	0.043 *
Use of endoscope (Yes vs. None)	−0.20 (−0.78, 0.39)	0.507	−0.22 (−0.90, 0.45)	0.513
Use of CVC (Yes vs. None)	0.47 (−0.32, 1.25)	0.242	−0.18 (−0.95, 0.60)	0.651
Use of FOLEY (Yes vs. None)	0.15 (−0.42, 0.72)	0.597	−0.37 (−0.93, 0.19)	0.189
Use of ventilator (Yes vs. None)	0.99 (0.46, 1.53)	<0.001 *	0.54 (−0.18, 1.25)	0.139

*: The mean difference is significant at the 0.05 level. Note: β is an unstandardized coefficient (converted by natural log). The coefficient that affects the length of hospitalization was adjusted for other variables (such as MDRO, age, sex, sample, previous ward, last departure, nutrition score, antibiotics, controlled medication, and *PsA* drugs), MRSA drugs, use of catheters, use of endoscopes, use of CVC, use of FOLY, and use of respirators).

**Table 3:** One-way ANOVA of log LOS for different anti-drug MDROs.

	Sum of Squares	Degrees of Freedom	Mean Sum of Squares	*F*	Significance (*p*)
Between Group	21.665	4	5.416	3.412	0.018
Within Group	58.741	37	1.588		
Sum	80.406	41			

**Table 4:** Table of post hoc tests of log LOS of different anti-drug MDROs.

Dependent Variable	MDRO ^a^		Mean Difference	Standard Error	Significance (*p*)	Post-Hoc Test
Log LOS	1	2	−1.24785	0.86948	0.725	
		3	−1.21241	1.15022	0.890	
		4	0.29371	0.82943	0.998	
		5	−1.35252	0.78011	0.563	
Log LOS	2	1	1.24785	0.86948	0.725	
		3	0.03545	1.01025	1.000	
		4	1.54156	0.62093	0.211	
		5	−0.10467	0.55333	1.000	
Log LOS	3	1	1.21241	1.15022	0.890	
		2	−0.03545	1.01025	1.000	
		4	1.50612	0.97599	0.668	
		5	−0.14012	0.93444	1.000	
Log LOS	4	1	−0.29371	0.82943	0.998	
		2	−1.54156	0.62093	0.211	5 > 4
		3	−1.50612	0.97599	0.668	
		5	−1.64623 *	0.48800	0.037 *	
Log LOS	5	1	1.35252	0.78011	0.563	
		2	0.10467	0.55333	1.000	
		3	0.14012	0.93444	1.000	5 > 4
		4	1.64623 *	0.48800	0.037 *	

*: The mean difference is significant at the 0.05 level. Scheffe’s post hoc test; ^a^: 1: VRE; 2: MRSA; 3: CREC. KP; 4: CRAB; 5: CRPA.

## Data Availability

The data presented in this study are available on request from the corresponding author. The data are not publicly available due to privacy restrictions.
